# The Core Social Fears Scale for Adolescents: Psychometric appraisal based on community and clinical samples

**DOI:** 10.1007/s00787-025-02824-4

**Published:** 2025-08-04

**Authors:** Paula Vagos, Diana Vieira Figueiredo, Marina Cunha

**Affiliations:** 1 Universidade de Aveiro, William James Center for Research, Aveiro, Portugal; 2 Center for Research in Neuropsychology and Cognitive Behavioral Intervention – CINEICC, Faculty of Psychology and Educational Sciences, University of Coimbra, 3000-115 Coimbra, Portugal; 3 Instituto Superior Miguel Torga, 3000-132 Coimbra, Portugal

**Keywords:** Social fears, Adolescence, Assessment, Factorial validity, Convergent validity

## Abstract

This study presents the Core Social Fears Scale for Adolescents (CSFS-A), developed to assess the Interaction, Performance, and Observation social fears that may be core to social anxiety in adolescence. This measure also assesses General Avoidance of social situations. A school-based sample of 3009 adolescents (53.7% female; age range: 12–19 years) and a clinical sample of 162 adolescents diagnosed with social anxiety disorder (70.4% female; age range: 15–18 years) provided information on anxiety experienced and avoidance engaged in during in social events; some participants also reported on another measure of social anxiety and fears of negative and positive evaluation. Confirmatory factor analyses supported a three-factor model reflecting correlated core social fears, as well as a unidimensional model for general avoidance, as applied to community and clinical participants. These models were invariant based on sex, age-groups (early, middle, and late adolescence), and clinical status. All measures had at least good internal consistency values. Evidence of convergent validity was found based on correlations between the scale’s measures and measures of social anxiety and fear of negative and positive evaluation. The Core Social Fears Scale for Adolescents proved to be a psychometrically robust measure for the assessment and characterization of the adolescents’ experience of social anxiety.

**Supplementary Information:**

The online version contains supplementary material available at 10.1007/s00787-025-02824-4.

## Introduction

Social anxiety refers to the discomfort experienced when anticipating or participating in social events where one may be subjected to the scrutiny of others. It may be felt in varying intensities, including normative levels that help individuals focus and cope with social demands, to mild or moderate intensities that are, nevertheless, transient and situation-specific [42]. In its most intense and persistent form, the individual may be diagnosed with Social Anxiety Disorder (SAD), which refers to the marked, persistent, and impairing fear experienced by the individual when facing social or performance situations. This often leads to such situations being avoided or experienced with extreme discomfort, often involving the use of various safety-seeking behaviors [3]. In addition to its affective (i.e., anxiety) and behavioral (i.e., avoidance and/or safety behaviors) characteristics, social anxiety has also been consistently associated with cognitive features, including the fear of being negatively (i.e., appearing unfavorably and undesirable to others) or positively (i.e., appearing favorably and thus as a potential threat to others) evaluated in social situations [25, 65].

Adolescents (i.e., individuals aged 12 to 20 years; [20]) are particularly vulnerable to experiencing social anxiety [8], due to the significant transitions they undergo at biological, individual, and social levels [34]. Previous studies suggest that adolescent girls, compared to adolescent boys, report higher levels of social anxiety [4, 7]. These authors further propose that such differences may be explained by gender-related roles internalized throughout development. Females may be socialized to construct an image of themselves as more interdependent [4] and as individuals whose social missteps are more strictly monitored and scrutinized [7]. In both cases, females may consequently become more reactive to their perceived social status.

Adolescence represents a continuous process of biological, emotional, social, and cognitive development, which is often conceptualized as comprising three stages: early adolescence (approximately 11 to 13 years), mid-adolescence (approximately 14 to 16 years), and late adolescence (approximately 17 to 19 years; [53]). Although relatively few studies have examined the expression of social anxiety across these stages, existing research has shown a general trend of decreasing levels of social anxiety [43, 48] and social avoidance [44] from early to mid- and late adolescence. This trend may reflect the heightened importance early adolescents place on being connected with peers who share similar interests, behaviors, or attitudes, combined with emotion regulation and complex thinking abilities that are still in early stages of development. Such factors may make them more prone to emotional arousal and outbursts [53]. Accordingly, difficulties in emotion regulation have been reviewed as contributing to how individuals respond in anticipation of, during, and following a feared social event [30]. In contrast, mid-adolescents, while still valuing interpersonal connections, begin to use these relationships as a basis for forming self-identity, whereas late adolescents tend to have developed a more stable sense of self within intimate and supportive relationships [53].

Despite age-related differences in the intensity of social anxiety, previous studies have reported prevalence rates for SAD during adolescence ranging from 8.3% [52] to 9.4% [2]. SAD typically follows a chronic course [6], potentially because social anxiety often goes unnoticed and/or is associated with reluctance to seek professional help [41]. Nevertheless, most adolescents experiencing SAD appear willing to receive treatment if it is offered [2], highlighting the importance of routinely screening for social fears in a private and non-threatening manner. This can be achieved through self-report questionnaires, particularly those that assess situational contexts known to elicit social fears as outlined in the DSM-5, namely, social interactions, being observed by others, or performing in front of others (criterion A; [3]).

Considering psychopathology from a situational perspective allows for a better understanding of fear or anxiety, as its intensity often depends on what the situation represents [26]. Skocic et al. [57] further suggested that diagnosing social anxiety should involve assessing whether fears are present/absent across three core situational dimensions: social interactions, being observed, and performing in front of others. These assumptions are supported by findings from both adult [13] and youth samples [32].

Using a large North American sample of individuals aged 18 years and older diagnosed with SAD, Cox et al. [13] conducted an exploratory factor analysis on 13 items representing feared social situations and found evidence supporting a three-factor model measuring social interaction fears, observation fears, and public speaking fears. The same authors further replicated this model in another independent sample of SAD-diagnosed adults using confirmatory factor analysis. The relevance of these three core social fears has also been demonstrated in clinically referred youth (aged 8–15 years) who met criteria for SAD, even when it was not their primary anxiety disorder [32]. Specifically, the authors conducted exploratory factor analyses on 23 items taken from the Anxiety Disorder Interview Schedule [56] related to social situations in which youth may experience fear and/or show avoidance. Results showed that these items were better represented by a three-factor solution reflecting the three core social fears: performance, observation, and interaction [32].

Alternatively, regarding avoidance behaviors, Skocic et al. [57] proposed that they should be considered in relation to their intensity, rather than their typology or the situational contexts in which they occur. This proposal was also supported by the empirical findings of Kodal et al. [32], who found that the same 23 items taken from the Anxiety Disorder Interview Schedule [56], when rated in relation to avoidance, were better represented by a single factor applicable across diverse social situations. The authors suggest that avoidance may not be situationally bound but rather reflect a general behavioral tendency, whereas social fears pertain to specific and situationally defined domains [32].

However, most self-report instruments available for adolescents were not designed with the specific purpose of assessing the three core and situation-based social fears found to characterize adolescents’ experience of social anxiety [32]. This is true for those instruments that have been reviewed by Tulbure et al. [58] to be empirically useful for screening social anxiety symptoms [i.e., the Social Phobia and Anxiety Inventory (SPAI; [10]), the Social Anxiety Scale for Adolescents (SAS-A; [33], and the Social Phobia Inventory (SPIN; [31])] or for detecting change in symptoms following interventions [i.e., SAS-A and the Liebowitz Social Anxiety Scale for Adolescents (LSAS-A; [40])].

Both the SPAI [10] and the SPIN [31] do not differentiate between the specific situational triggers of social anxiety; the former considers cognitive, physiological, affective, and behavioral symptoms of social anxiety as a single construct, while the latter addresses fear, avoidance, and physiological symptoms. The SAS-A [33] includes only one situational dimension (e.g., new social situations), and the LSAS-A [40] considers social anxiety in relation to both interaction and performance. None of these instruments assess the “being observed” core social fear, and all were adapted from instruments originally developed to assess adult experiences of social anxiety.

Alternatively, the Social Anxiety and Avoidance Scale for Adolescents (SAASA; [3]) was specifically developed to address the social events and stimuli that may characterize adolescents’ experiences across a variety of commonly encountered social situations (e.g., performance in formal social settings). It assesses both the intensity of anxiety or fear felt in social events and the frequency of avoidance of those events across six social dimensions (i.e., Interaction in New Social Situations, Interaction with the Opposite Sex, Being Observed by Others, Performance in Formal Situations, Assertive Interaction, and Eating and Drinking in Public). Previous research has demonstrated its psychometric soundness among both early and mid-adolescents [15] and late adolescents [61]. Although the six dimensions assessed by the SAASA (see Instruments section) are theoretically grounded, they do not fully align with the conceptualization of social anxiety in adolescence based on three core social fears [3, 18, 32, 57]. Still, given its situational focus, the SAASA appears to be an optimal foundation from which to refine and validate a measurement model that directly addresses the three core social fears identified as central to subtyping social anxiety in adolescence [18, 32, 57].

### Current work

The current work relies on the theoretical proposal that social fears should be considered in relation to specific social situations namely social interaction, being observed, and performing in front of others [57], a proposition that has found empirical support in the results by Kodal et al. [32]. Skocic et al. [57] also proposed that avoidance should be assessed in terms of intensity (i.e., from little or no impairment to severe impairment), rather than being situation specific. This conceptualization was also reflected in Kodal et al. [32] finding of a single avoidance component. We intend to refine (in one community subsample) and subsequently test (in another community subsample) the psychometric properties of a three-core social fears measurement model (i.e., Observation, Interaction, and Performance) applied to the items of the SAASA. The cross-validation of this model in adolescents diagnosed with SAD will also be examined. Based on previous findings, we expect the three-core social fears model to provide a good fit for describing social anxiety in our samples and to demonstrate adequate internal consistency [32]. A secondary objective is to investigate a General Avoidance measurement model. Drawing on previous exploratory findings in a clinically referred sample [32], we hypothesize that a one-factor model will be a good fit for representing General Avoidance.

This work will also analyze the internal structure validity, internal consistency, and measurement invariance of these measurement models across sex and age groups (i.e., early-, mid-, and late-adolescence), as these stages of adolescence involve different interpersonal and intrapersonal challenges and transitions [53]. Given that the SAASA has been found applicable to early-, mid- [15], and late-adolescents, and its scores were invariant by sex [61], we expect to observe both sex- and age-based measurement invariance. Females are expected to report more intense social fears [4, 7], whereas all three core social fears and general avoidance are expected to decrease with age [43, 44]. Convergent validity of the core social fears and avoidance measures will also be investigated in relation to another measure of social anxiety, as well as to measures of fear of negative and positive evaluation. We expect to find evidence of convergent validity for the proposed measurement models, mirroring previously found associations with other social anxiety measures [15] and with fear of negative and positive evaluation [65]. Finally, given the continuous nature of social anxiety [42], we expect the measurement models to be invariant across community and clinical samples, with the latter reporting higher levels of social fears and general avoidance.

## Method

### Participants

#### Community sample

The community sample was recruited from previous studies with specific aims, namely to characterize social anxiety and its associations with individual temperament, family, and peers [15, 17], and to investigate the relationship between social anxiety and assertiveness [60]. Participants were recruited from schools located in the central and northern regions of Portugal, after obtaining institutional authorizations (i.e., the General Directorate of Education and the administrations of the participating schools). Schools were randomly selected to ensure balanced representation of public and private institutions, urban and rural settings, and schools with varying positions in the national school rankings. Each school was contacted by a member of the research team, who presented the study and requested the school’s collaboration by inviting three classes from each school year to complete the research protocol. Prior to any contact with students, informed consent was obtained from parents or legal guardians of participants under 18 years old. Additional inclusion criteria were applied post hoc: participants had to be aged between 12 and 19 years and not enrolled in the special education needs system.

A community sample of 3030 was initially recruited, of which 21 participants were excluded due to missing values completely at random (MCAR $\chi$2(116) = 127.68, *p* = 0.22). So, the complete community sample was composed of 3009 adolescents who filled in the SAASA, aged 12 to 19 years old. Among them, 53.7% were female, and 58.7% were attending secondary school (Table 1). To accomplish our research goal of investigating measurement invariance of the Core Social Fears Scale for Adolescents (see Instruments section) across age-groups, we categorized the complete sample into: early adolescents aged 11–13 years old (*n* = 341, 22.8%), mid-adolescents ages 14–16 years old (*n* = 741, 49.5%), and late adolescents aged 17–19 years old (*n* = 416, 27.8%).

**Table 1:** Sociodemographic characterization of community and clinical participants

	Community sample			Clinical sample (*n* = 162)
	Complete (*n* = 3009)	Subsample 1 (*n* = 1511)	Subsample 2 (*n* = 1498)	
Age [M(SD)]	15.08 (1.86)	15.04 (1.88)	15.12 (1.84)	15.81 (1.02)
Sex [*n*(%)]				
Male	1392 (46.3)	681 (45.1)	711 (47.5)	48 (29.6)
Female	1617 (53.7)	830 (54.9)	787 (52.5)	114 (70.4)
School level [*n*(%)]				
3rd school level	1242 (41.3)	622 (41.2)	620 (41.4)	18 (11.1)^a^
Secondary school	1766 (58.7)	889 (58.8)	877 (58.5)	144 (88.9)

^a^All participants in the 3rd school level in the clinical sample attended the 9th grade

Also, for the purpose of refining and validating the instrument under analysis, the complete sample was randomly split into two subsamples of similar sizes (Table 1), using the split sample function in IBM SPSS software. Participants in these two subsamples had similar mean ages [*t*_(3007)_ = 1.14, *p* =.26] and were evenly distributed by biological sex [$\chi$^2^_(1)_ = 1.73, *p* =.19], school level [$\chi$^2^_(1)_ = 0.20, *p* =.89], and age-groups [$\chi$^2^_(2)_ = 1.05, *p* =.59]. Subsample 1 (*n* = 1511) was used for the refinement of the measurement models and subsample 2 (*n* = 1498) was used for the psychometric evaluation of the refined models (see Data Analyses).

Additional information was collected from some adolescents in the complete community sample and used for convergent validity analyses. A group of 2188 adolescents aged 12 to 18 years old (M = 14.60, SD = 1.86) completed the SAASA and the Social Anxiety Scale– Adolescents (SAS-A). Most of these participants were female (*n* = 1109, 50.7%), attended the third school level (*n* = 1242, 56.8%), and were early adolescents (*n* = 1125, 54.1%). A separate group of 391 participants filled in the SAASA, the Brief Fear of Negative Evaluation (BFNE) and the Fear of Positive Evaluation Scale (FPES). They were aged 14 to 19 years old (M = 16.50, SD = 1.12); most were female (*n* = 275, 70.3%) and late adolescents (*n* = 197, 50.4%). All participants in this group were attending secondary school.

#### Clinical sample

The clinical sample was collected as part of two previous studies on the efficacy of psychological interventions for adolescent SAD [5, 16, 21] were invited for an individual diagnostic assessment using the Mini-International Neuropsychiatric Interview for Children and Adolescents [55], Portuguese version by [50] to establish a diagnosis of SAD. Participants who received a primary diagnosis of SAD, did not present psychotic symptoms, and were not receiving psychological intervention at the time were invited to participate in the studies. Those who consented completed the self-report protocol, and there were no missing values.

The complete clinical sample included 162 participants who received a primary diagnosis of SAD (Table 1), aged 14 to 18 years old. Most were female (70.4%), attended secondary school years (88.9%), and were mid-adolescents (66.0%).

As part of the pre-intervention protocol applied in one of the studies from which this sample was drawn [5, 21], a group of adolescents within this clinical sample completed the SAS-A in addition to the SAASA (*n* = 92), most of whom were male (*n* = 72, 78.3%) and mid-adolescents (*n* = 66, 71.7%), aged 15 to 18 years old (M = 15.99, SD = 0.87). All attended secondary school. Another group of 70 participants completed the BFNE and the FPES, in addition to the SAASA. They were aged 14 to 18 years old (M = 15.59, SD = 1.15); most were male (*n* = 42, 60%), mid-adolescents (*n* = 41, 58.6%), and attended secondary school (*n* = 52, 74.3%). These groups of clinical participants were used in convergent validity analyses.

### Instruments

All instruments were applied in their Portuguese version.

#### Core Social Fears Scale for Adolescents (CSFS-A)

The CSFS-A for adolescents uses the 30 items that compose the SAASA [15]. Twenty-eight of those items comprised the anxiety measure (i.e., items 5 and 11 were excluded) and 26 items comprised the avoidance measure (i.e., items 9, 10, 13, and 29 were excluded; [61]). The CSFS-A used those same items and proposed a new way of organizing them based on the three core social fears and on general avoidance.

The SAASA [15] is a self-report questionnaire assessing common social situations where adolescents may experience fear/anxiety and avoidance. Its original version includes 34 items answered twice for evaluating the intensity of anxiety felt (i.e., SAASA-anxiety) and intended avoidance (i.e., SAASA-avoidance) when facing social situations. Its version adapted to late adolescents [61] consists of 30 items, also answered twice for the same two subscales. Each item (e.g., “Going to a party given by a colleague”) is answered on a five-point Likert scale, ranging from *none* (1) to *very much* (5) for anxiety, and from *never* (1) to *almost always* (5) for avoidance.

Subscales are composed of the same six dimensions, though they include different items for the anxiety and avoidance measures: Interaction in New Social Situations, Interaction with the Opposite Sex, Being Observed by Others, Performance in Formal Situations, Assertive Interaction, and Eating and Drinking in Public. Most dimensions showed acceptable internal consistency values for the anxiety scale in the original version (i.e., α ≥ 0.73), except for α = 0.67 for Assertive Interaction and α = 0.61 for Eating and Drinking in Public. Alternatively, three dimensions showed acceptable internal consistency values for the avoidance measures (α ≥ 0.70), while α = 0.59 for Being Observed by Others, α = 0.55 for Assertive Interaction, and α = 0.54 for Eating and Drinking in Public fall below this threshold [15]. All internal consistency values were at least acceptable for the SAASA version adapted to late adolescents: α ≥ 0.82 for anxiety dimensions and α ≥ 0.71 for avoidance dimensions [61]. The SAASA showed good 5-week test–retest reliability, the capacity to discriminate adolescents with SAD from adolescents with other anxiety disorders and without psychopathology, and construct validity in relation to other social anxiety, anxiety, and depression measures [15], as well as to negative social thoughts [61].

For information on the psychometric properties information of the CSFS-A using the current samples, see the results section.

#### Social Anxiety Scale– Adolescents (SAS-A; [33], Portuguese version by [16])

The SAS-A is a self-report questionnaire assessing adolescents’ subjective experiences of social anxiety. It includes 22 items answered on a five-point Likert scale indicating how much each item “is true for you” (1 = ‘not at all’ to 5 = ‘all the time’). Because 4 of the 22 items are filler items (e.g., “I like to read”), only the remaining 18 items are organized into three subscales: Fear of Negative Evaluation (e.g., “I worry about what others say about me”), Social Avoidance and Distress in New Situations (e.g., “I get nervous when I meet new people”), and Generalized Social Avoidance and Distress (e.g., “I feel shy even with peers I know very well”). In the original study, all subscales presented at least acceptable internal consistency values (*α* ≥ 0.76; [33]).

The Portuguese version of the SAS-A also had at least acceptable internal consistency values for all subscales (α ≥ 0.71), acceptable 4-week test–retest reliability, and construct validity with respect to other social anxiety, anxiety, and depression measures [16]. In the current work, the SAS-A subscales achieved acceptable internal consistency values for both the community (α > 0.74) and clinical (α > 0.71) samples.

#### Brief Fear of Negative Evaluation (BFNE; [51], adapted Portuguese version by [12])

The BFNE uses eight straightforward items to assess fear of negative evaluation. Each item (e.g., “I am afraid others will not approve of me”) is answered on a five-point Likert scale ranging from *not at all like me* (1) to *very much like me* (5). Previous work found excellent internal consistency values with adult clinical (α = 0.92; [66]) and non-clinical samples (α = 0.94; [51]), convergent validity in relation to measures of social anxiety [51, 66], discriminant validity in relation to measures of anxiety sensitivity and depression, ability to discriminate for the presence/absence of SAD, and sensitivity to cognitive-behavioral group treatment effects [66].

The Portuguese version of the BFNE was applied to adolescents by Correia et al. [12], who proposed to adapt four of its items to more specifically relate to fear of negative evaluation. Thus constituted, the measure showed evidence for a one-factor structure, excellent internal consistency (α = 0.92), and construct validity in relation to social anxiety and avoidance [12]. In the current work and using its Portuguese version with specified items, the BFNE also attained excellent internal consistency values for the community (α = 0.93) and the clinical (α = 0.90) samples.

#### Fear of Positive Evaluation Scale (FPES, [67], Portuguese version by [62, 63])

The FPES consists of 10 items of which 8 are used to measure fear of positive evaluation. Previous works showed evidence on the measures’ good internal consistency values with adult non-clinical (α = 0.80; [67]) and clinical samples (α = 0.83; [68]), convergent validity in relation to measures of social anxiety and fear of negative evaluation, and discriminant validity in relation to generalized anxiety, worry, depression, and quality of life [67, 68]. The use of the Portuguese version of the FPES with an adolescent sample further confirmed its one-factor internal structure, good internal consistency (α = 0.87), and construct validity in relation to social anxiety and avoidance [63]. That Portuguese version of the FPES achieved at least acceptable internal consistency values for the current community (α = 0.86) and clinical (α = 0.77) samples.

### Data analyses plan

Data analyses followed a three-step approach: refinement of the measurement models in community participants, psychometric analyses of the refined models in community participants, and psychometric analyses of the refined models in clinical participants. This theory-driven (e.g., [57]) approach was based on previous literature that sustained a confirmatory (rather than exploratory) strategy, and on the intention to grasp overarching theoretical constructs (rather than explore specific characteristics of our samples).

Model refinement used community subsample 1 (*n* = 1511) to achieve the most parsimonious and internally homogenous measures for each core social fear and for general avoidance. Concerning core social fears, items referring to each core social were tested as independent one-factor solutions (see Results). Those measurement models were considered acceptable when achieving a Standardized Root Mean Square Residual (SRMR) value ≤ 0.08 combined with either a Root Mean Square Error of Approximation (RMSEA) value ≤ 0.06 or with a Comparative Fit Index (CFI) value ≥ 0.95 [27]. Internal consistency based on Cronbach Alpha values were deemed acceptable if ≥ 0.70 [47]. After these models were considered acceptable, a three-correlated factor measurement model representing the three core social fears was tested and judged on acceptability based on the same criteria. As for General Avoidance, all items were entered into the analysis as one single factor, with the same acceptability criteria being applicable.

Then, community subsample 2 (*n* = 1498) was used to examine the fit of the three-factor fear and one-factor avoidance measurement models and assess their measurement invariance based on sex and on age-groups. The same criteria stated above were used to judge the acceptability of the models. Measurement invariance analyses followed a forward approach [19] and considered: (1) configural invariance (i.e., the measurement models achieved acceptable fit indicators for each group separately); (2) metric invariance (i.e., the fit indicators didn’t worsen meaningfully, when comparing a non-equality model with a loading-equality model); (3) scalar invariance (i.e., the fit indicators didn’t worsen meaningfully, when comparing the loading and intercept-equality model with the loading-equality model). A non-meaningful change in the model was judged based on the guidelines by Chen [9]. Convergent validity was also analyzed within this step, using all available community participants (see participants).

Finally, the clinical sample (*n* = 192) was used to cross-validate the same three-social fears and one-avoidance measurement models. A subgroup of community participants was selected to be of similar size to the clinical sample and served to test for measurement invariance. The same criteria for judging the acceptability and measurement invariance of the measurement models detailed above were applied. Convergent validity was also investigated using available clinical participants.

Data taken from the full sample (i.e., subsample 1, subsample 2 and clinical sample– *n* = 3171) was not multivariate normal for the 28 items addressing anxiety (Mardias’ skewness z = 33654,91, *p* <.001; Mardias’ kurtosis = 234.32, *p* <.001) nor for the 26 items addressing avoidance (Mardias’ skewness z = 49420.35, *p* <.001; Mardias’ kurtosis = 264.12, *p* <.001). So, the Maximum Likelihood Robust estimator was used for measurement model and measurement invariance analyses conducted in MPlus v7.4 [46]. Likewise, convergent validity analyses were based on non-parametric correlation analyses (i.e., spearman rho). Internal consistency and convergent validity analyses relied on the IBM SPSS Statistic 26 software.

## Results

### Refinement of the measurement model within community subsample 1

#### Anxiety measure

Three separate one-factor CFA were conducted (i.e., one for each core social fear) to refine each measurement model: Observation core social fear used 9 items pertaining to the Observation by others and to Eating and drinking in public dimensions of the SAASA; Performance core social fear considered 4 items composing Performance in formal social events dimension of the SAASA; Interaction core social fear including 15 items referring to the Interaction in new social situations, Assertive interaction, and Interaction with the opposite sex dimensions of the SAASA. Fit indices for the Performance measure were acceptable (Table 2); items had *λ* ≥ 0.69. Alternatively, fit indices were below acceptability for Observation and Interaction.

**Table 2:** Fit indicators for CFA and measurement invariance analyses applied to the anxiety

	$\chi$^2^	df	RMSEA	90% CI for RMSEA	CFI	SRMR
Model refinement (*n* = 1511)						
One-factor Observation model (9 items)	85.83	20	.047	.037;.057	.96	.031
Refined one-factor Observation model (8 items)	261.49	27	.076	.068;.084	.88	.049
One-factor Performance model (4 items)	15.88	2	.068	.039;.100	.99	.019
One-factor Interaction model (9 items)	120.72	27	.048	.039;.057	.95	.032
Refined one-factor Interaction model (15 items)	906.40	90	.077	.073;.082	.83	.059
Three-factor core social fears measurement model (21 items)	587.75	186	.038	.034;.041	.94	.035
Model cross-validation to community subsample (*n* = 1498)						
Three-factor measurement model (21 items)	695.18	186	.043	.039;.046	.92	.038
Measurement invariance across sex						
Male sample (*n* = 711)	388.49	186	.039	.034; 0.045	.91	.046
Female sample (*n* = 787)	520.81	186	.048	.043;.053	.92	.042
Unrestrictive model	899.44	372	.044	.040;.047	.91	.044
Metric invariance model	980.69	390	.043	.039;.047	.91	.049
Scalar invariance model	1047.75	408	.046	.042;.049	.89	.052
Partial scalar invariance model	986.56	405	.044	.040;.047	.90	.050
Measurement invariance across age-groups						
Early adolescents (*n* = 341)	301.46	186	.043	.034;.051	.89	.052
Early adolescents (*n* = 741)	525.19	186	.050	.045;.055	.90	.046
Late adolescents (*n* = 416)	367.71	186	.048	.041;.056	.93	.047
Unrestrictive model	1200.17	558	.048	.044;.052	.91	.048
Metric invariance model	1238.42	594	.047	.043;.050	.91	.052
Scalar invariance model	1371.25	630	.049	.045;.052	.90	.054
Partial scalar invariance model	1352.16	627	.047	.044;.051	.90	.053
Model cross-validation to clinical sample (*n* = 162)						
Three-factor measurement model (21 items)	264.72	186	.052	.037;.065	.95	.043
Measurement invariance across community and clinical groups						
Community subgroup (*n* = 155)	269.87	186	.054	.039;.068	.89	.062
Unrestrictive model	537.71	372	.053	.043;.063	.93	.053
Metric invariance model	559.09	390	.052	.042;.062	.93	.063
Scalar invariance model	580.20	408	.052	.042;.061	.93	.066

All $\chi$2 values were significant at *p* < 0.001. *df* = degrees of freedom, *RMSEA* = Root Mean Square Error of Approximation, *CFI* = Comparative Fit Index, *SRMR* = Standardized Root Mean Square Residual

Observation and Interaction measures were refined by eliminating redundant items: in each pair of items with high error covariances as proposed by modification indices, the item with the highest loading value was retained. This resulted in an 8-item refined Observation measure (i.e., item 2 was eliminated) and a 9-item refined Interaction measure (i.e., items 6, 8, 12, 17, 18, and 19 were eliminated) that acceptably fitted the data (Table 2). All items had *λ* ≥ 0.51 for both measures. No items were taken out of the Performance measure because it had achieved acceptable fit.

The initial Performance measure and the refined Observation and Interaction measures were investigated via CFA as a three-correlated factor measurement model, which was a good fit for the data (Table 2), with *λ* ≥ 0.52 and α = 0.79 for Observation, *λ* ≥ 0.58 and α = 0.77 for Performance, and *λ* ≥ 0.43 and α = 0.80 for Interaction (Supplementary Material - Table A).

#### Avoidance measure

A one-factor model with 26 items composing the avoidance measure was not a good fit for the data. It was refined based on eliminating redundant items (i.e., items 2, 4, 6, 8, 15, 18, 22, 23, 24, and 25). This resulted in a 16-item one-factor measurement model that adequately fitted the data (Table 3), with λ ≥ 0.36 (Supplementary Material– Table B) and α = 0.79.

**Table 3:** Fit indicators for CFA and measurement invariance analyses applied to avoidance

	$\chi$^2^	df	RMSEA	90% CI for RMSEA	CFI	SRMR
Model refinement (*n* = 1511)						
One-factor Avoidance (21 items)	2093.19	299	.063	.060;.066	.71	.061
Refined one-factor Avoidance (16 items)	396.81	104	.043	.039;.048	.88	.040
Model cross-validation to community subsample (*n* = 1498)						
One-factor Avoidance (16 items)	419.48	104	.045	.041;.050	.87	.041
Measurement invariance across sex						
Male sample (*n* = 711)	238.31	104	.043	.036;.050	.86	.047
Female sample (*n* = 787)	301.34	104	.049	.043;.056	.86	.047
Unrestrictive model	538.53	208	.046	.041;.051	.86	.047
Metric invariance model	562.49	223	.045	.040;.050	.86	.052
Scalar invariance model	682.21	238	.050	.046;.054	.81	.056
Partial scalar invariance model	593.52	233	.045	.041;.050	.85	.052
Measurement invariance across age-groups						
Early adolescents (*n* = 341)	160.39	104	.040	.027;.052	.89	.051
Early adolescents (*n* = 741)	250.08	104	.044	.037;.050	.88	.045
Late adolescents (*n* = 416)	252.41	104	.059	.049;.068	.84	.058
Unrestrictive model	669.72	312	.048	.043;.053	.86	.050
Metric invariance model	732.62	342	.048	.043;.053	.86	.058
Scalar invariance model	825.69	372	.049	.045;.054	.83	.061
Partial scalar invariance model	759.61	368	.046	.042;.051	.85	.059
Model cross-validation to clinical sample (*n* = 162)						
One-factor Avoidance (16 items)	161.72	104	.059	.040;0.076	.93	.052
Measurement invariance across community and clinical groups						
Community subgroup (*n* = 155)	152.15	104	.055	.034;.073	.83	.066
Unrestrictive model	313.80	208	.057	.043;.069	.90	.059
Metric invariance model	351.98	223	.060	.048;.072	.88	.083
Partial metric invariance model	338.19	222	.057	.045;.069	.89	.076
Scalar invariance model	372.91	237	.060	.048;.072	.88	.082
Partial scalar invariance model	363.11	236	.058	.046;.070	.88	.081

All $\chi$2 values were significant at *p* < 0.001. *df* = degrees of freedom, *RMSEA* = Root Mean Square Error of Approximation, *CFI* = Comparative Fit Index, *SRMR =* Standardized Root Mean Square Residual

### Cross-validation of the measurement models to community subsample 2

#### Anxiety measure

The three-factor measurement model was a good fit for the data taken from subsample 2 (Table 2), with *λ* ≥ 0.53 and α = 0.79 for Observation, *λ* ≥ 0.61 and α = 0.79 for Performance, and *λ* ≥ 0.48 and for α = 0.80 Interaction (Supplementary Material– Table A).

##### Measurement invariance and latent mean comparisons across sex

The 21-item three-correlated factor model was a good fit to the data taken separately from male and female participants, pointing to configural invariance (Table 2). Full metric invariance was also established (*Δ*RMSEA = −0.001, *Δ*CFI = −0.002, *Δ*SRMR = 0.005). Partial scalar invariance was achieved after relaxing the intercepts of items 13, 21, and 27 (*Δ*RMSEA = 0.001, *Δ*CFI = −0.007, *Δ*SRMR = 0.001). Female participants scored significantly higher than male participants on Observation (latent mean = 0.404, *p* <.001), Performance (latent mean = 0.497, *p* <.001), and Interaction (*p* = 0.361, *p* <.001). The same was seen in descriptive values (Table 4).

**Table 4:** Descriptive values for core social fears in the community and clinical participants

	Core social fears			General avoidance
	Observation	Performance	Interaction	
Community sample (*n* = 1498)	13.24 (5.06)	9.09 (3.68)	18.05 (5.98)	28.46 (8.11)
Community sample by sex				
Male (*n* = 711)	11.96 (4.14)	8.19 (3.26)	16.82 (5.44)	26.87 (7.43)
Female (*n* = 787)	14.40 (5.53)	9.90 (3.85)	19.16 (6.23)	29.89 (8.43)
Community sample by age-groups				
Early adolescence (*n* = 549)	13.04 (4.57)	8.65 (3.59)	18.37 (5.65)	29.23 (8.25)
Middle adolescence (*n* = 533)	13.43 (5.09)	9.14 (3.67)	18.20 (6.01)	28.47 (7.87)
Late adolescence (n = 416)	13.06 (3.74)	9.36 (3.74)	17.52 (6.15)	27.81 (8.39)
Clinical Sample (*n* = 162)	20.18 (8.49)	12.77 (4.62)	25.65 (8.50)	38.36 (13.93)

Values are presented as M(SD). The scores for the community subgroup used for latent mean comparison with the clinical sample were M = 13.67 (SD = 5.21) for Observation, M = 9.75 (Sd = 3.99) for Performance, M = 18.91 (SD = 6.40) for Interaction, and M = 29.39 (SD = 8.20) for General Avoidance

##### Measurement invariance and latent mean comparisons across age groups

The 21-item three-correlated factor fitted acceptably to early-, mid-, and late-adolescents separately, defining configural invariance. Full metric invariance was determined (*Δ*RMSEA = −0.001, *Δ*CFI = −0.000, *Δ*SRMR = 0.004), followed by partial scalar invariance after allowing the intercepts of items 13, 16 and 23 to differ across age-groups (*Δ*RMSEA = 0.000, *Δ*CFI = −0.006, *Δ*SRMR = 0.001). Latent mean comparisons showed no significant differences between age groups; see descriptive values by age groups in Table 4.

#### Avoidance measure

The 16-item one-factor measurement model was a good fit for the data taken from subsample 2 (Table 3), with λ ≥ 0.37 (Supplementary Material– Table B) and α = 0.79.

##### Measurement invariance and latent mean comparisons across sex

The one-factor measurement model was an acceptable fit for males and females separately, thus establishing configural invariance. Full metric invariance was subsequently achieved (*Δ*RMSEA = −0.001, *Δ*CFI = −0.004, *Δ*SRMR = 0.005), followed by partial scalar invariance, after relaxing the intercepts of items 11, 12, 21, 17 and 27 (*Δ*RMSEA = 0.000, *Δ*CFI = −0.009, *Δ*SRMR = 0.000; see Table 3). Latent mean comparisons show that female participants (latent mean = 0.220) scored significantly higher than male participants (*p* <.001), alike descriptive data (Table 4).

##### Measurement invariance and latent mean comparisons across age groups

The one-factor measurement model fitted acceptably to all age groups, indicating configural invariance. Full metric invariance was then established (*Δ*RMSEA = −0.002, *Δ*CFI = −0.003, *Δ*SRMR = 0.008), followed by partial scalar invariance, after allowing the intercepts of items 11, 12, and 19 to vary across all age-groups (*Δ*RMSEA = 0.000, *Δ*CFI = −0.01, *Δ*SRMR = 0.001). Latent mean comparisons indicate no significant differences; descriptive values across age groups are depicted in Table 4.

### Cross validation of measurement models to a clinical sample

#### Anxiety measure

The three-factor core social fears model was a good fit for the clinical sample (Table 2), with *λ* ≥ 0.62 and α = 0.90 for Observation, *λ* ≥ 0.75 and α = 0.87 for Performance, and *λ* ≥ 0.65 and α = 0.90 for Interaction (Supplementary Material– Table A).

##### Measurement invariance across community and clinical groups

Invariance was tested between the complete clinical sample (*n* = 162) and a randomly selected subgroup of community participants (5% of the complete community sample; *n* = 155) so that sample sizes across clinical status were balanced. The three-factor model was a good fit for the community subgroup, thus defining configural invariance (Table 2). Full metric (*Δ*RMSEA = −0.001, *Δ*CFI = −0.001, *Δ*SRMR = 0.01) and full scalar invariance (*Δ*RMSEA = 0.000, *Δ*CFI = −0.002, *Δ*SRMR = 0.003) were also established. Latent mean comparisons further show that, in comparison with community participants, clinical participants scored significantly higher on Observation (latent mean = 0.81, *p* <.001), Performance (latent mean = 0.71, *p* <.001), and Observation (latent mean = 0.86, *p* <.001), alike descriptive values (Table 4).

#### Avoidance measure

The 16-item one-factor measurement model fitted the clinical sample acceptably (Table 3) with λ ≥ 0.52 (Supplementary Material—Table B) and α = 0.92.

##### Measurement invariance across community and clinical groups

The 16-item one-factor model fitted acceptably to the same community subgroup, thus establishing configural invariance (Table 2). Only partial metric (*Δ*RMSEA = 0.000, *Δ*CFI = −0.01, *Δ*SRMR = 0.017) and scalar (*Δ*RMSEA = 0.001, *Δ*CFI = −0.001, *Δ*SRMR = 0.005) invariance was found, after relaxing the loading of item 28 and the intercept of item 9, respectively. Participants from the clinical group had a significantly higher latent mean score (0.69, *p* <.001) than the community subgroup, like descriptive measures (Table 4).

#### Evidence of convergent validity within community and clinical participants

The three core social fears correlated positively and significantly with each other (*r*_*s*_ between 0.59 and 0.76, all *p*s <.001), and with general avoidance (*r*_*s*_ between 0.55 and 0.74, all *p*s <.001). Core social fears and general avoidance correlated positively with social anxiety and with fear of negative and positive evaluation. These correlation values were always statistically significant for community participants. For clinical participants, core social fears and general avoidance did not associate significantly with the fear of negative evaluation measure of the SAS-A, and Performance did not associate significantly with the generalized social avoidance and distress measures of the SAS-A (Table 5).

**Table 5:** Construct validity evidence based on association with external variables, for community and clinical participants

	Core social fears			Avoidance
	Observation	Performance	Interaction	
Social Anxiety Scale– Adolescents				
FNE	.37^**^|.17^+^	.31^**^|.06^+^	.38^**^|.15^+^	.31^**^|.14^+^
SAD-New	.48^**^| 34^*^	.44^**^|.28^*^	.51^**^|.35^*^	.49^**^|.33^*^
SAD-General	.41^**^|.44^**^	.31^**^|.14^+^	.39^**^|.36^**^	.39^**^|.46^**^
Fear of Negative Evaluation	.50^**^|.38^*^	.39^**^|.40^*^	.50^**^|.44^**^	.45^**^|.38^*^
Fear of Positive Evaluation	.44^**^|.47^**^	.38^**^|.39^*^	.45^**^|.52^**^	.45^**^|.42^**^

*FNE* = Fear of Negative Evaluation, *SAD-New* = Social Avoidance and Distress of New Situations, *SAD-General* = Generalized Social Avoidance and Distress. Values are presented as *r*_*s*_ for the community sample| *r*_*s*_ for the clinical sample

^**^ *p* < 0.001,^*^ *p* < 01,^+^ *p* > 0.05

## Discussion

Social anxiety refers to discomfort felt when facing social events, and it may be experienced in diverse levels of intensity, from an everyday and adaptive occurrence to an excessive and impairing experience [42]. Hence, and notwithstanding the relevance of intrapersonal variables as explicative factors (e.g., self-focused attention; [35, 38]), social anxiety seems to be, per definition, linked to situational aspects that define the core social stimuli one may fear. This is considered when performing a diagnosis for SAD [i.e., criteria A referring to social anxiety occurring when facing social interactions, when being observed, and when performing in front of others; [3]]. Likewise, considering social anxiety as pertaining to those three core social fears has been proposed as useful in understanding and subtyping social anxiety experiences [32, 57]. However, self-report instruments are not aligned with this perspective. The current work intended to propose and investigate the psychometric properties of the Core Social Fears Scale for Adolescents (CSFS-A). This measure uses 21 items to address Observation (8 items), Performance (4 items), and Interaction (9 items) social fears. For the wording of the items composing each measure see Table A in the Supplementary Material.

Observation and Interaction fears included items referring mostly to interacting with peers within school contexts. Performance, in turn, included items revolving around academic performance within classrooms. SAASA, from which items were taken, had been developed to address situations particularly relevant to adolescence, and so the importance of schools and their socialization agents (i.e., peers and teachers) is to be expected. Also, the relevance of these items aligns with schools being crucial contexts for development and well-being in adolescence [22]. Of note, the Interaction fear overall reflected assertive interaction, with items pertaining to interaction in new social events or with the opposite sex being mostly excluded in the refinement process. Items included in the Interaction measure focus on questioning or requesting a change in others’ attitude or behavior, which may be particularly challenging [1]. Social anxiety was previously associated with assertive deficit [60], which may hinder the social performance of socially anxious individuals [45].

Core social fears were invariant across biological sex. Female participants presented overall higher scores, alike previous findings [39, 49], which concurs with the CSFS-A likely addressing social fears representative of social anxiety. Core social fears were also similarly applicable to community adolescents of diverse age groups. No noticeable differences were found between those groups, though descriptive values show a very slight increase for performance fears and decrease for interaction fears as adolescents grow older. Previous works have found that social anxiety overall decreased from younger to older adolescents [43, 48], though specific core social fears were not considered. When differentiating core social fears, and considering a developmental perspective as proposed by Salmela-Aro [53], it seems plausible that late adolescents worry more about their performance as they prepare for the transition to emerging adulthood and, for example, to higher education, while they worry less about interactions because they have developed intimate and supportive peer-groups.

Core social fears were also as relevant to characterize social fears in community participants as in participants with SAD, who present higher scores of those social fears. This applicability of the same measurement model across community and clinical levels of social anxiety adds evidence to the relevance of the social anxiety continuum [42, 54]. So, the trajectory from normative to pathological social fears may be grounded in non-situational variables. Other variables may better serve to understand and intervene with those suffering from social anxiety. Some of those variables (e.g., negative cognitions, self-focused attention, the practice of safety-behaviors) may be taken from Clark and Wells [11] model of social anxiety, which has been proven effective for explaining [37, 64] and intervening in adolescent SAD [36, 38, 59]).

Besides social fears, avoidance of social events is also a criterium for diagnosing SAD [3]. Our findings align with the previous proposition that avoidance consists of a general behavioral tendency towards various social situations [32]. Our items seem to address overt avoidance, and not subtle avoidance/safety-seeking behaviors. Both have been proposed by Skocic et al. [57] as relevant aspects underlying social anxiety, and their role in sustaining social anxiety has been supported by empirical findings [35, 38, 44]. It may be that situational aspects are not relevant for overt avoidance precisely because situations are avoided completely and so are not considered in their specificities. Safety-seeking behaviors, in turn, may be multifaceted constructs (e.g., avoiding others’ attention on oneself, investing in coming across in the best possible way, or concealing physiological symptoms; [14]). Some dimensions of safety-seeking behaviors will likely be more relevant for situations depicting specific core situational features (e.g., coming across well may be relevant to Interaction and Performance events, but not as much to Observation situations). This analysis has not, to our knowledge, been conducted.

Like core social fears, the general avoidance measure was invariant across biological sexes, age groups, and community/clinical status. Also, latent mean comparisons show that female participants and participants with SAD scored higher than males and community participants, respectively; no age-based differences were found. On the one hand, the finding that female participants and participants with SAD report more general avoidance aligns with social anxiety and avoidance frequently appearing together, either as diagnostic guidelines [3, 57] or as associated constructs [15]. Our own correlation analyses further corroborate the interlink between these constructs. As for the finding that general avoidance is similar across age-groups, it mirrors the stable trajectory of avoidance experienced by most adolescents [44]. In practical terms, our participants may have limited ability to avoid school contexts– that were targeted in the items– due to their age.

Current results favor the convergent validity of the three core social fears and of general avoidance within community participants. As expected, higher core social fears and higher general avoidance were associated with higher social anxiety in general and in new social situations [15], and with fear of negative and positive evaluation [63]. As such, subtyping social anxiety in relation to core social fears and to a general avoidance tendency seems to be (another) useful way of understanding social anxiety, as it relates to relevant factors, proposed not only to have a role in characterizing [57] but also in explaining [65] social anxiety. Still, within our clinical sample, we unexpectedly found no significant correlations between core social fears and measures form the SAS-A. To our knowledge, the SAS-A had seldomly been used with clinical samples [see Garcia-Lopez et al., [23] for an exception], and not in relation to core social fears. Items on the SAS-A were developed based on theory, rather than on symptomatology, and so may be less apt do accurately identify and characterize clinical SAD samples [28]. While further work is needed, it may be that our clinical sample did not relate with the items of the SAS-A and so their responses did not align with their report on fears associated with specific eliciting contexts.

Though findings support the psychometrics of the CSFS-A, some limitations should be noted. We adopted a confirmatory and a statistical approach to data analyses and model refinement, based on the theoretical assumption and empirical data pointing to three core social fears [32, 57]. As such, the current proposal may have left out some particularities of items’ associations. Our clinical sample was older than our community sample. Though we found age-related measurement invariance and no meaningful age-related differences in core social fears or in general avoidance, it would be useful to verify if this pattern holds for clinical samples; given previous findings by Kodal et al. [32], age-based differences may be evident for clinical samples. We relied solely on self-reported data and addressed only convergent validity. Self-reported information should be further validated by observation, measures of physiological arousal, and/or clinical appraisal. Finally, though our sample is large, and our findings seem framed by previous literature, participants came from the north and central regions of Portugal only and so findings may not generalize to samples outside of Portugal. A more geographically and linguistically diverse sample would increase confidence in the generality of current findings.

Adolescence is marked by an increased importance attributed to peers, with whom adolescents spend most of their time, and who represent opportunities for marking out one’s identity and social role [29]. By limiting access to those opportunities, social anxiety may significantly impair adolescents’ well-being and overall development. In addition to the intensity of social anxiety, it seems relevant to understand the situational aspects that may represent specific vulnerabilities and intervention priorities [32]. Having a simple, private, and quick way of assessing those vulnerabilities may, for example, inform how to custom behavioral experiments within a Cognitive Therapy [11] framework. About general avoidance, it may be helpful to promote engagement with social situations that align with one’s values, irrespective of those values being applicable to one or to various social situations, as assumed by Acceptance and Commitment Therapy [24]. The Core Social Fears Scale for Adolescents has proven psychometrically sound enough to be taken forward in designing, evaluating, and disseminating the best practices for psychological intervention in adolescent SAD.

## Supplementary Information

Below is the link to the electronic supplementary material.

Supplementary file1 (DOCX 49 KB)

## Data Availability

The datasets generated and analyzed during the current study may be made available by the corresponding author without undue reservation.
