# Supplementary material for: The Core Social Fears Scale for Adolescents: Psychometric appraisal based on community and clinical samples
Source: Eur Child Adolesc Psychiatry. 2025 Aug 4;35(1):191–204. doi: 10.1007/s00787-025-02824-4 (PMC12916510; doi:10.1007/s00787-025-02824-4)
Supplement: Supplementary file 1 — Supplementary file1 (DOCX 49 KB) [file 787_2025_2824_MOESM1_ESM.docx]

| Table A: Loading values for items accessing the three Core Social Fears | | | | |
| --- | --- | --- | --- | --- |
|  |  | Community sample | | Clinical sample  (n = 162) |
|  |  | Subsample 1  (n = 1511) | Subsample 2  (n = 1498) |  |
| Observation | |  |  |  |
|  | 1. Eating in public | .52 | .53 | .62 |
|  | 3. Going to a party given by a colleague | .53 | .53 | .76 |
|  | 21. Making exercises during gym class | .59 | .59 | .77 |
|  | 22. Changing in the shower room | .53 | .55 | .72 |
|  | 27. Participating in a group sport | .63 | .63 | .77 |
|  | 28. Crossing the hall, corridors or going to the canteen/school bar when it is full of students | .62 | .66 | .77 |
|  | 29. Participating in school parties | .54 | .55 | .70 |
|  | 30. Answering back to a colleague that is trying to make fun of me | .59 | .54 | .79 |
| Peformance | |  |  |  |
|  | 4. Reading aloud in front of the class | .58 | .61 | .75 |
|  | 23. Having an oral test or exam | .70 | .70 | .76 |
|  | 25. Being asked to solve a problem on the blackboard | .71 | .72 | .83 |
|  | 26. Taking the initiative of asking a question or for an explanation in a class or meeting | .71 | .73 | .83 |
| Interaction | |  |  |  |
|  | 7. Talking to someone I don’t know very well | .56 | .57 | .66 |
|  | 9. Expressing disagreement or disapproval to a colleague I don’t know very well | .58 | .59 | .76 |
|  | 10. Making eye contact with someone I don’t know very well | .58 | .56 | .71 |
|  | 13. Performing, for the first time, a new task or role in front of colleagues | .67 | .65 | .79 |
|  | 14. Saying “no” to a colleague who has asked me to do something I don’t want to | .51 | .54 | .65 |
|  | 15. Mingling in a group where there are mainly people from the opposite sex | .57 | .53 | .59 |
|  | 16. Asking someone for a favor | .50 | .48 | .79 |
|  | 20. Asking a colleague to change a way of behaving which annoys me | .56 | .56 | .78 |
|  | 24. Complaining when someone tries to jump the queue | .43 | .50 | .65 |

| Table B: Loading values for items accessing General Avoidance | | | |
| --- | --- | --- | --- |
|  | Community sample | | Clinical sample  (n = 162) |
|  | Subsample 1  (n = 1511) | Subsample 2  (n = 1498) |  |
| 1. Eating in public | .38 | .45 | .65 |
| 3. Going to a party given by a colleague | .43 | .46 | .65 |
| 5. Writing while being observed | .37 | .38 | .52 |
| 7. Talking to someone I don’t know very well | .55 | .48 | .71 |
| 11. Expressing my feelings to the person I like | .46 | .42 | .58 |
| 12. Being alone with a colleague from the opposite sex | .51 | .54 | .51 |
| 14. Saying “no” to a colleague who has asked me to do something I don’t want to | .36 | .39 | .51 |
| 16. Asking someone for a favor | .70 | .41 | .71 |
| 17. Making a compliment to someone of the opposite sex | .54 | .50 | .61 |
| 19. Talking with other colleagues | .50 | .53 | .68 |
| 20. Asking a colleague to change a way of behaving which annoys me | .44 | .42 | .68 |
| 21. Making exercises during gym class | .45 | .38 | .58 |
| 26. Taking the initiative of asking a question or for an explanation in a class or meeting | .48 | .50 | .63 |
| 27. Participating in a group sport | .48 | .49 | .70 |
| 28. Crossing the hall, corridors or going to the canteen/school bar when it is full of students | .46 | .48 | .71 |
| 30. Answering back to a colleague that is trying to make fun of me | .45 | .37 | .64 |
